# Decisions, decisions, decisions. A qualitative exploration of decision-making in performance support leaders

**DOI:** 10.3389/fspor.2025.1664191

**Published:** 2025-11-13

**Authors:** P. J. Wilson, Gregory Roe, John Kiely

**Affiliations:** 1 Setanta College, Department of Education, Limerick, Ireland; 2 Department of PE and Sports Sciences, University of Limerick, Limerick, Ireland; 3 Carnegie School of Sport, Leeds Beckett University, Leeds, United Kingdom

**Keywords:** judgment, sports, support staff, leadership, teamwork

## Abstract

Despite the assumed importance of decision-making in competitive sport environments, little is known about how performance support leaders (PSLs) make decisions within their professional contexts. This study aimed to explore how PSLs in high-performance sport approach decision-making tasks and understand the factors that influence their decisions. Using a pragmatic philosophy, fifteen PSLs working in performance sport, working across a range of countries and sports, were interviewed using a rigorously developed semi-structured interview protocol. Thematic analysis identified three key themes: emotional intelligence competencies, experience, and organizational structuring. The PSLs placed significant importance on having the necessary emotional intelligence to self-regulate and work cooperatively with others and having the necessary experience to develop the context-specific knowledge and skills required to make effective decisions. PSLs also emphasised the value of collaborative approaches when making difficult decisions, and the need for an organisational structure enhancing and enabling decision-making through clear systems, processes, and departmental structures. PSLs referenced individual and group reflective practices as key promoters of learning from previously made decisions. However, most PSLs have not undertaken any formal decision-making training. Future research should evaluate how these factors' impact decision-making effectiveness in high-performance sport, through formal assessment of processes and outcomes. These findings may help establish a framework for developing evidence-based interventions serving to enhance decision-making effectiveness. The identified elements of emotional intelligence, experiential knowledge, and organizational structures represent critical leverage points that may enhance leadership practices and driving measurable improvements in performance outcomes.

## Introduction

Decision-making, choosing a particular course of action from a set of alternative options (1), is a critical component of performance support staff responsibilities (2, 3). Decisions often involve input from multiple stakeholders within the team or organisation, which may include practitioners such as coaches, medical staff, physical performance staff and even players (3). In high-performance sport environments, these decisions directly impact athlete development, injury management, and competitive outcomes. Making decisions requires careful consideration of potential outcomes of each course of action before making an appropriate selection (2). However, accurate judgement and decision-making in dynamic, fast-moving sporting contexts is difficult (4).

The human cognitive system has inherent limitations preventing effective processing of the large volumes of complex information (1, 4). This fundamental gap between our mental processing capacity and the informational processing requirements need to effectively navigate complex decision-making demands leads to a reliance on intuitive mental shortcuts, or heuristics. Heuristics simplify complex decisions to manageable portions (5). Heuristics offer efficiency in many contexts (6). Yet, in many others, heuristics introduce cognitive biases (7) that significantly detract from decision-making proficiency (8–10). This inherent tension, between cognitive limitations and decision-making complexity, represents a fundamental challenge for PSLs.

The context in which a decision is made, whether largely predictable or inherently unpredictable, fundamentally influences decision quality (4). This contextual distinction is particularly relevant for Performance Support Leaders (PSLs), who coordinate multidisciplinary teams in sport environments.

High validity contexts feature regular, predictable patterns that decision-makers can learn to recognize through experience. This pattern recognition provides meaningful information that enhances forecast accuracy (11, 12). Early rehabilitation represents such a context, where practitioners focus on promoting healing, managing pain, and reestablishing movement within controlled parameters (13). Here, measurable indicators such as pain levels, strength assessments, and biomechanical markers provide clear evidence of progress (14, 15).

Conversely, low validity contexts lack reliable, discernible patterns. Repeated exposure to these irregular and uncorrelated situations does not enable pattern recognition and consequently does not improve predictability. In these environments, making decisions based on historical experiences often confounds rather than enhances decision-making effectiveness (11, 12). PSLs frequently face such low validity contexts when making complex decisions—for example, when predicting and allocating short and long-term resources to maximize program performance (16). These situations present specific challenges as they require PSLs to navigate uncertainty while maintaining decision confidence, despite the absence of clear patterns and reliable indicators.

Notably, recent research in other complex decision-making domains has resulted in the development of evidence-based strategies for enhancing decision-making outcomes (17–20). Such strategies include bias mitigation training, teamwork skills training, and the implementation of rigorous monitoring of decision outcomes (7, 21, 22). Given the importance of decision-making in high-performance sport, such strategies may be beneficial. Yet, despite this potential value, there is limited research examining how these approaches might be applied within sport-specific contexts and adapted to meet the unique demands faced by PSLs. Even though recent research has suggested that evidence-based decision-making models and strategies should be adopted in applied sport practice to enhance decision quality (3).

Currently, however, it remains unclear how PSLs typically make decisions within applied sport contexts. Research in this area could help identify potential opportunities for improving professional sports decision-making. Addressing this knowledge gap is particularly important given the increasing complexity and professionalisation of performance support in contemporary sport environments. Accordingly, the aim of this study was to explore how PSLs in high-performance sport approach decision-making while better understanding the factors that influence their decisions.

More specifically, this research focused on the following three objectives:

1. Investigate PSLs' perceptions of the importance of decision-making in their roles, and their perceptions of how they might enhance decision-making effectiveness
2. Examine PSLs’ current decision-making processes, methods for tracking outcomes, and their strategies for navigating obstacles to effective decision-making
3. Uncover PSL’s perceptions of the characteristics of effective decision-makers in high-performance sport.

These objectives, collectively, provide a comprehensive framework for understanding not only how decisions are currently made in high-performance sport environments, but also how decision-making processes could be systematically enhanced through evidence-based approaches and targeted professional development.

## Materials & methods section

### Experimental approach to the problem

#### Research philosophy

The current study was undertaken with the primary objective of producing practically meaningful knowledge. In other words, the study was philosophically underpinned by pragmatism (23). In contrast to traditional research paradigms (e.g., positivism and constructivism), pragmatism does not take a particular ontological or epistemological stance. Instead, pragmatists argue research should provide practically useful solutions to an *a priori* identified problem (24).

In this study, we aimed to understand PSLs' perceptions of different facets of decision-making to ultimately determine if, and where, decision-making processes in high performance sport contexts might be enhanced. To do so, a pragmatic process of inquiry was implemented, whereby the methods employed were perceived to be effective for addressing the research aims (24). Specifically, a descriptive qualitative approach with semi-structured interviews was implemented to allow the acquisition of in-depth information from participants, while maintaining the focus of the study. This methodological approach is consistent with previously published qualitative studies in sports medicine journals examining complex decision-making phenomena (25).

#### Design

This descriptive qualitative study employed semi-structured interviews to collect rich, contextual data on PSLs' decision-making processes in their professional contexts. Interviews lasted 45–60 min and were conducted via video conferencing software (Microsoft Teams). Video conferencing facilitated both verbal and non-verbal communication. The interview guide was developed based on a review of decision-making literature and refined through pilot testing with two experienced sport performance professionals not included in the final sample. Ethical approval was obtained from the Education and Health Sciences Research Ethics Committee at the University of Limerick, Ireland [approval number: 2021_01_13_EHS (RA)]. Consent was obtained from all participants prior to participating in the semi-structured interviews.

#### Subjects

Purposive criterion sampling was used to recruit participants for this study (26). Criteria were as follows: (a) a performance support staff member with a minimum of 5 years' experience in a leadership position with significant decision-making responsibilities, (b) currently working in high-performance sports. Participants were identified in two ways. Firstly, the research study was advertised on social media (Twitter/X) and Linkedin, the professional networking platform, for an 8-week period. Secondly, the authors explored potential candidates within their professional networks (authors have a collective experience of over 40 years working in high-performance sport and a diverse network of professional contacts). Participants meeting the criteria were contacted by the authors.

When participants provided referrals, these referrals were also contacted if inclusion criteria were satisfied. Of the 22 PSLs who were initially approached, 15 agreed to participate (68% response rate). The final sample included 15 participants (12 male, 3 female). Each participant's role title, sport in which they work, and years of experience are presented in Table 1.

**Table 1:** Participant descriptors.

Role	Sport	Years experience
Head of Performance Support	Olympic multi-Sport	16 years
Head of Strength & Conditioning	Professional Rugby Union	15 years
Performance Support Lead	Olympic Sport	18 years
Performance Support Lead	Olympic Sport	21 years
Lead Strength & Conditioning	Professional Rugby Union	14 years
Head of Athletic Performance	Professional Rugby Union	16 years
Performance Director	Professional Rugby Union	20 years
Head of Performance	Professional Football/Soccer	20 years
High Performance Lead	National Basketball Association (NBA)	10 years
Senior Director of Athletic Performance	National Football League (NFL)	13 years
Head of Rugby	Professional Rugby Union	27 years
National Programmes Coach Developer	Professional Rugby Union	8 years
Head of Medical Services	Professional Football/Soccer	14 years
High-Performance Director	Rugby Union	34 years
Physical Performance Lead	Professional Football/Soccer	17 years

#### Procedures

The research study utilised semi-structured interviews (see Appendix 1). The interview guide was developed using the framework proposed by Kallio et al. (27). An initial guide was constructed in line with previous research that identified strategies used by, and characteristics of good forecasters (18, 28, 29). Interview questions were designed to investigate PSLs' perception of the importance of decision-making in their roles (1 question); how PSLs approach difficult decision-making scenarios (1 question); PSLs' perceptions of their decision-making processes individually and in teams (1 question); PSLs views on training strategies to enhance decision-making effectiveness (3 questions); investigate if, and by what means, PSLs monitor the process and outcome of decisions (3 questions); PSLs' perceptions of obstacles that hinder effective decision-making (1 question); and how PSLs characterise good decision-making (1 question). After initial design, the interview guide was sent to a panel of experts (3 individuals with minimum of 5 years' experience in a leadership role in high performance sport with experience of decision-making research). They were asked to assess and provide feedback on whether the questions would effectively capture the facets of decision-making under investigation without leading participants to a particular answer, and whether the structure of the questionnaire was coherent, and the content of each question was clear and understandable. The experts provided feedback, which the authors evaluated using available evidence, to support or reject their recommendations. This process continued until both the panel of experts and the authors were satisfied that the interview guide content was suitable for capturing the relevant information. The final semi-structured interview consisted of 12 distinct inquiries. Each question was designed to delve into a critical facet of the participants' professional roles, perceptions of decision-making processes, and the nuances surrounding effective decision-making in the context of high-performance sport leadership. To elaborate, one question was centred on the participants' specific roles and their perspectives on the intricacies of decision-making. Another query aimed to unveil potential obstacles that may obstruct the decision-making process. The questions further explored the strategies employed by the interviewees when confronted with particularly challenging decision-making scenarios. Additional questions probed the attributes and qualities deemed requisite for adept decision-makers, the dynamics of teamwork and collaborative decision-making, comprehensive approaches to tracking and evaluation, as well as considerations for education and training. A final question, categorised as “any other business,” offered time for participants to share unanticipated or unexplored insights. The interview guide is presented in Appendix Table A1.

#### Analyses

Each interview was recorded and transcribed verbatim to ensure accuracy and fidelity to the participants' responses. To ensure trustworthiness of the data analysis process, we employed Lincoln and Guba's criteria for qualitative rigor: credibility, transferability, dependability, and confirmability (30). Thematic analysis was conducted using Microsoft Excel (version 16.73) and Microsoft Word was used for data organization, coding structure development, and theme visualization. Inductive thematic analysis was used to identify patterns of meaning within the data set (31). Key stages in this analysis were familiarisation, coding, theme development, refining, naming, and write up. In the first stage, authors PJW and GR familiarised themselves with the data to ensure both had a comprehensive understanding of all the interviews. In stage 2, PJW immersed himself in the data, systematically coding content of interest and associated data extracts from the transcripts. Codes were organized and grouped according to their conceptual similarities to form potential themes. Once finished, GR reviewed each transcript and critically evaluated the codes. Themes were then developed, refined, and named through an iterative process of discussion between researchers, with particular attention to how they addressed the three research objectives. Researcher reflexivity was maintained throughout the analysis process, with the research team acknowledging their backgrounds in sport science and potential biases during regular reflexive discussions held after each phase of coding and theme development. All data were securely stored in password-protected files on institutional servers in accordance with data protection regulations.

## Results

Results from the semi-structured interviews are presented in Table 2. The analysis yielded 18 themes that align with the three primary research objectives. These themes are organized into three core areas: (1) Perceptions of decision-making importance and enhancement opportunities, (2) Decision-making processes, outcome tracking, and obstacle navigation, and (3) Characteristics of effective decision-makers, as perceived by PSLs.

**Table 2:** Questions, themes, theme descriptions, and codes from experienced coaches decision-making perceptions of importance, processes, obstacles, and enhancing strategies in professional sporting contexts.

Theme	Description	Code
A) Question: ‘Tell me about your role? And, how important is DM to your role?’		
Decision-making is a critical component of role responsibilities	Performance support leaders emphasised the significance of decision-making in their roles, regarding it as a fundamental determinant of success. They recognised that effective decision-making underpins the achievement of desired outcomes and the attainment of performance goals.	Decision-making is pivotal to their roles
B) Question: ‘Talk to me about team decision-making and what comes with it?/What is a leader's role in team decision-making?’		
Well-developed emotional intelligence competencies	Performance support leaders emphasised the importance of leveraging their interpersonal skills to foster collaboration within teams	Relationship management skills Empower team members
Organisational structuring: transparent inter- and intra-departmental structures	Performance support leaders explicitly described elements of an organisational structure that were required to facilitate team decision-making	Facilitate open lines of communication Ensure roles and responsibilities are clear Develop systems and processes to maximise collaboration
C) Question: ‘Are you tracking decision-making performance? ‘Do you review strategies that may be directly or indirectly linked to decision-making?’ ‘How do you learn from your decision-making (good and bad)?’		
Organisational structuring: direct and/or indirect methods for tracking decisions	Experienced coaches utilise both direct and indirect strategies to track and monitor the progress of decisions made and the decision-making process	Multiple methods of feedback
Reflective practice	Reflective practice was presented as a method of learning from decisions, with practitioners engaging in self- and group-reflective practice.	Self-reflective practice Group-reflective practice
D) Question: ‘When faced with a difficult decision, what do you do/how do you arrive at a decision?’		
Experience	Participants repeatedly emphasised the importance of having experience when making difficult decisions, implicitly implying that relevant contextual knowledge is gained through experience	Experience is an antecedent to the contextual knowledge required to make difficult decisions
Team decision-making	Gathering the perspectives of different members of the performance support team was described as a key step when making difficult decisions.	Gather the perspectives of team members
Well-developed emotional intelligence competencies	Performance support leaders referenced aspects of emotional intelligence, particularly self- and social-awareness as important skills to have when making difficult decisions.	Self-awareness Social awareness
E) Question: ‘What are the characteristics of good-decision-making in your environment/context?’		
Big picture thinkers	Participants communicated how they acquired a vast amount of contextual knowledge through their experiences, enabling them to assess the broader context surrounding a decision. Participants described how extensive experience in professional sport environments is required to develop contextual knowledge, which augments decision-making.	An ability to assess and interpret the context and bigger picture around a decision.
Team decision-making	Gathering the perspectives of different members of the performance support team was described as a key step in the decision-making process.	Gather the perspectives of team members
Well-developed emotional intelligence competencies	Emotional intelligence, particularly self-awareness and social awareness, were referenced as important skills of good decision-makers.	Self-awareness Social awareness Relationship management skills
Organisational structuring: systems and processes	Participants explicitly referenced the need for appropriate systems and process to support decision-making in their contexts.	Implement systems and procedures to help guide the decision-making process.
F) Question: ‘What are the biggest obstacles you are currently seeing to decision-making in elite sport?’		
Inexperience	Performance support leaders pointed out that Inexperienced practitioners lack contextual knowledge, attenuating their decision-making abilities.	Lack of experience and knowledge
Underdeveloped emotional intelligence competencies	Participants implicitly and explicitly referenced a lack of self-awareness as an obstacle to good decision-making, particularly with respect to ego.	Poor self-awareness Ego
Insufficient organisational structures	Participants described scenarios in which elements of an organisational structure were lacking and resultantly hindered decision-making.	A lack of systems and processes
Environmental pressures	Participants explicated specific factors relating to their working environments that negatively affected good decision-making.	Uncertainty Time constraints
G) Question: ‘Is Decision-making a trainable skill?’ ‘Have you ever had formal decision-making education?’		
Decision-making is a learnt skill	Decision-making was perceived by performance support leaders to be a skill that can be developed over time. Furthermore, the importance of experiential learning and practice in improving decision-making abilities was highlighted.	Experiential learning and practice are perceived as critical to improving decision-making ability
Formal/targeted decision-making training is not commonly undertaken but is of interest	Participants emphasised that most performance support staff have not undertaken any formal decision-making training, apart from those with medical training, but expressed a willingness to engage in formal training initiatives.	Only medically trained practitioners have been exposed to formal decision-making training, normally within their academic training Practitioners would welcome formal decision-making training

### Perceptions of decision-making importance and enhancement opportunities

In response to the question “tell me about your role, and how important is decision-making to your role” (Table 2A), PSLs discussed the importance and constant nature of decision-making and referred to decision-making as a key determinant of success. Specifically, all 15 PSLs suggested the importance of decision-making. This is represented in the theme “Decision-making as a critical component of role responsibilities”, which is supported by the following data extracts:

**Table:** 

Participant	Extract
Participant 6:	‘..I would say the main part of that role is making decisions.'
Participant 8:	‘..so decision making absolutely critical to, to day-to-day operation..'

PSLs also expressed openness to enhancing their decision-making capabilities through both experiential learning and formal training, recognizing decision-making as a skill that develops over time.

### Decision-making processes, outcome tracking, and obstacle navigation

When questioned about team decision-making and a leader's role in team decision-making (Table 2B), the following two themes emerged, (1) Well-developed emotional intelligence competencies, and (2) Organizational structuring.

The theme *Well-developed emotional intelligence competencies* highlight how PSLs emphasised the importance of relationship management skills to build trusting relationships and empower people to encourage effective collaboration within their teams.

**Table:** 

Participant	Extract
Participant 8:	'So for me, it's really important to have people that you can trust. People that you, have enough knowledge that you can empower them.'
Participant 1:	‘..empower staff to make decisions,..'

To further support effective team decision-making, PSLs underscored the need for a clear organisational structure. This facilitates enhanced communication, clarity in roles, and through the utilisation of systems and processes, maximises collaboration.

**Table:** 

Participant	Extract
Participant 12:	‘.. collaboration done really well is where those silos are really minimised through connection and, and creating time and opportunities for those connections,..'
Participant 3:	‘..we have..’ - ‘..processes or methods of working that support collective interaction and appropriate decision making..'

When asked about how they monitor the process and outcomes of decisions (Table 2C), PSLs discussed both direct and indirect approaches. Such methods involved the use of ongoing communication and feedback loops, incorporating live in-the-moment feedback and hot-reviews, maintaining documentation for review, journal keeping, and conducting meetings to further inform the process.

**Table:** 

Participant	Extract
Participant 13:	‘..incorporate both written text and conversational feedback loops with everything we do..'
Participant 13:	‘..with simple decisions [..] we have a like a feedback loop in our day [..] Back end of the day, we have a wrap up meeting..'

Moreover, PSLs emphasised the importance of reflective practice, noting both group- and self-reflective skills as tools to track, review and learn from decision-making.

**Table:** 

Participant	Extract
Participant 4:	'we do review decisions we make [..] and we'll learn from it..'
Participant 9:	‘..every chance we get to reflect on a process and identify the decisions that were made [..] and [..] really critically analyse them.'

When questioned about navigating difficult decisions (Table 2D), PSLs described how they draw upon their experience, make use of team decision-making, and have well developed emotional intelligence competencies.

When discussing their experience, PSLs leverage the knowledge and expertise they have built over time to assess and interpret the bigger picture surrounding decisions.

**Table:** 

Participant	Extract
Participant 5:	‘.. two or three things that will feed into a decision being made. The most common one is always prior experience. Lean heavily on prior experience and I've been in this situation in the past. What have I done to be successful or to make the right decision? And what does that look like? And then you compare what's in front of you to that prior experience.'
Participant 4:	‘..you get more confident in your own ability to answer questions on the back of the fact that you are experienced and [..] you've seen a lot of different contexts across a lot of different environments.'

Additionally, PSLs harness the power of team-based decision-making to gather diverse perspectives further informing the process.

**Table:** 

Participant	Extract
Participant 4:	‘..you're also much more comfortable with asking for help in making that decision because you're more open to the fact that you don't know everything. You can't know everything but other people, and that's where I think the diversity of opinions is really helpful with coming to the right conclusion.'
Participant 14:	‘..obviously I look to get differences of opinion and also to consider the opinions and perceptions of others.'

PSLs also identified several obstacles to effective decision-making, including inexperience, underdeveloped emotional intelligence competencies, insufficient organizational structures, and environmental pressures. These obstacles align with their perspectives on effective processes, emphasizing the importance of overcoming such barriers through structured approaches and team collaboration.

### Characteristics of effective decision-makers

Following questioning on characteristics of good decision-makers (Table 2E), PSLs described good decision-makers as big picture thinkers; as individuals who have acquired a vast knowledge base through experience that enables them to assess and interpret the broader picture surrounding decisions.

**Table:** 

Participant	Extract
Participant 11:	'a good decision maker 1) is going to be well-rounded from a knowledge standpoint in essence, they can consider all the lenses, remain detached from their own lens, while making the decision, to not be influenced or biased.'
Participant 8:	‘..I can't underestimate the importance of experience in decision making. The more you know and the more experience you get, the easier decisions become and the faster that you can make them so, am, I think even the context around which people make decisions is really important.'

Additionally, PSLs perceived good decision-makers as individuals who utilise team decision-making.

**Table:** 

Participant	Extract
Participant 4:	‘..informed decision making is really important'
Participant 9:	‘..they consider different perspectives,..'

Moreover, PSLs identified good decision-makers as having well-developed emotional intelligence competencies. Specifically, they referred to self-awareness and social awareness.

**Table:** 

Participant	Extract
Participant 4:	‘..good decision makers can probably separate their biases driven by their own egos and use the objective information that they know to be true or false to then make a decision which might not align with their own biases, but it's informed.'
Participant 6:	‘..have to be able to be able to read people's non verbal communication as well, [..] so I've got to be able to read the room, read the body language as well.'

Finally, according to PSLs, good decision-makers consistently utilise decision-making systems and processes.

**Table:** 

Participant	Extract
Participant 4:	‘..very consistent in terms of decision-making processes from the perspective of meeting times, meeting structures, meeting outcomes,..'
Participant 9:	‘..the defining characteristic of good decision making, I would say it's recognising that it's about the process of making the decision, not necessarily the outcome.'

These characteristics of effective decision-makers directly inform and align with the strategies PSLs reported using in their own practice, demonstrating coherence between their stated ideals and professional approaches. The emphasis on experience, emotional intelligence, and structured processes reflects the integrated nature of decision-making in high-performance sport environments.

## Discussion

The overall aim of the present study was to explore how PSLs in high-performance sport address decision-making problems and to better understand the factors influencing their decisions. PSLs perceived decision-making as integral to their roles and crucial for achieving success. PSLs emphasised the importance of encouraging collaboration among team members, via emotional intelligence competencies and leveraging organisational structuring strategies.

While PSLs did not intentionally track decisions, both formal and informal strategies were incorporated into the decision-making process. Additionally, they highlighted the significance of self-reflection and group-reflection to extract valuable insights from decision-making outcomes. When faced with difficult decisions, PSLs relied on their experience and emotional intelligence competencies to effectively navigate challenging choices. PSLs identified experience, knowledge, and emotional intelligence competencies as key characteristics of good decision-makers and identified inexperience and time constraints as obstacles inhibiting good decision-making. Despite their lack of formal decision-making education, PSLs expressed openness to undertaking formal decision-making training. Notably, and in agreement with other recent research (22), PSLs recognised that enhancing decision-making skills and proficiencies requires ongoing deliberate practice and experiential learning.

Overall, four themes were common across multiple questions from the interviews. These were ‘Well-developed emotional intelligence competencies', ‘organisational structuring: transparent inter- and intra-departmental structures', ‘experience', and ‘team decision-making' (Table 2). These findings align with the three research objectives: (1) understanding the importance and enhancement of decision-making, (2) examining decision-making processes and obstacles, and (3) identifying characteristics of effective decision-makers. The interrelationships between these themes create a comprehensive framework for understanding decision-making in high-performance sport contexts.

### Decision-making importance and enhancement opportunities

#### Emotional Intelligence

Emotional intelligence is an umbrella term for the skills and competencies necessary for an individual to recognise, comprehend and manage their emotions, and the emotions of others (32). Based on Goleman's model of emotional intelligence (32), results illustrate the importance PSLs place on specific emotional intelligence competencies for decision-making (Tables 2B, D–F). Overall, self-awareness was referenced as important for navigating difficult decisions, and as a key characteristic of good decision-makers. In contrast, an insufficiency of self-awareness was deemed an obstacle to good decision-making. Self-awareness allows individuals to evaluate their emotional response to a decision-making scenario and subsequently to determine whether they are in the right frame of mind, and have the necessary information and knowledge, to effectively make the decision (33).

Additionally, social awareness and relationship management were also discussed by PSLs. Both were implicitly or explicitly referenced as important when faced with difficult decisions (Table 2D). Moreover, social awareness was perceived as a characteristic of good decision-makers (Table 2E). Within the social awareness/relationship management domain (Table 2B), building trusting relationships and supporting team decision-making were identified as core pillars. These skills enable PSLs to make decisions that are considerate of both individuals and organisational context; thereby increasing trust, reducing conflict, and promoting team performance (33, 34).

Notably, the primacy of emotional intelligence in our findings aligns with research in other high-stakes decision environments (9, 10). This line of research takes on particular significance in sport contexts, where interpersonal dynamics and pressure-filled scenarios are fundamental defining characteristics (4). Notably, this emphasis on emotional intelligence represents a departure from traditionally-focussed technical and tactical approaches to sport leadership. This reasoning emphasises the sophisticated interpersonal demands, contexts and relationship pressures placed on contemporary PSLs.

### Decision-making processes, tracking, and obstacle navigation

#### Organisational structuring

PSLs emphasised the importance of organisational structuring –including decision-making systems and processes and transparent inter- and intra-departmental structures– for supporting decision-making (Table 2). PSLs recognise the necessity of a leader's ability to structure their working environment is a defining characteristic of a good decision-maker (Table 2E). In contrast, inadequate organisational structuring is perceived as an impediment to good decision-making (Table 2F).

Decision-making within fast-moving, high-pressure sports ecosystems is complex and inherently difficult (3, 4). Establishing systems and processes to underpin decision-making in such environments –such as information quality control (35); defining decision types and establishing decision rules (36); allocating clear roles and responsibilities (37) and establishing clear channels of communication (16)– may improve decision-making effectiveness. Furthermore, prior work suggests that embedding research and development processes into the daily work of sports practitioners serves to also enhance performance-related decision-making outcomes (38). Notably, the organizational structures described by PSLs demonstrate how formal decision frameworks should be adapted to the unique tempo and pressures of high-performance sport. Unlike corporate settings where decision matrices may be standardized (20), sport requires frameworks sufficiently fluid to accommodate both the unpredictable nature of dynamic competition schedules and the unpredictable demands of competition and injury management (4, 14).

### Experience

Interviewed PSLs considered experience to be a critical attribute of good decision-makers (Table 2E). In contrast, inexperience was thought to impede good decision-making (Table 2F). Experience was viewed as an antecedent to the in-depth contextual knowledge required to make difficult and complex decisions (Table 2D). Such contextual information enables decision-makers to appreciate environmental subtleties, discern amongst nuanced options and subsequently select more contextually appropriate decision options (39, 40).

Furthermore, decision-making was viewed as a learned skill, primarily developed through experiential learning over time (Table 2G). Experiential learning requires the learner to be embedded in the context in which specific skills are required; requires the learner to actively deliberate on future outcomes; to have ample opportunity to critically reflect and problem solve, and to be continually exposed to novel decision-making problems (41). These learning exposures are demonstrably more effective than traditional forms of learning (42), and is the training approach typically used by top geopolitical forecasters (28).

Nevertheless, decision-making can also be enhanced through specifically focussed learning approaches, such as short online learning modules in probabilistic reasoning and decision scenario-based training (29, 43). Such learning approaches were not discussed by PSLs, yet can drive decision-making enhancements. Notably, the emphasis on experience reveals an important tension in high-performance sport decision-making. Specifically, while experiential knowledge is clearly valued, the low-validity contexts PSLs often face (44) means that past experiences may not reliably predict future outcomes. This paradox underlines the need for both deep domain expertise and adaptable decision frameworks explicitly recognising the limitations of experience-based heuristics (5, 6).

### Characteristics of effective decision-makers

#### Team decision-making

Under the theme of team decision-making PSLs reported that gathering the perspectives of different members of the performance support team was a key step in the decision-making process, particularly when making difficult decisions (Table 2D). Furthermore, PSLs viewed this approach as critical to good decision-making in high-performance sport contexts (Table 2E). Supporting these beliefs, previous research demonstrates that effective teamwork and shared decision-making contribute to better team decision-making and decision outcomes in other complex contexts, such as geopolitical forecasting (22). Moreover, research investigating performance leadership and management processes in performance sport demonstrates the importance of teamwork for programme success (16, 45).

PSLs also discussed aspects of their roles that augmented team decision-making (Table 2B). Under the theme of well-developed emotional intelligence competencies (Table 2B), PSLs emphasised the importance of leveraging relationship management skills to build trusting relationships and empower individuals within their team. Such factors were identified as crucial for team performance (46), particularly with respect to team decision-making accuracy (47). Furthermore, under the theme ‘Organisational structuring’ (Table 2B), PSLs explained elements of an organisational structure that should be put in place to encourage effective team decision-making. These included facilitating open lines of communication, ensuring roles and responsibilities are clear among team members, and developing support systems and processes to maximise collaboration. These findings corroborate findings from previous research in high-performance sport (16, 37).

The integration of team decision-making with both emotional intelligence competencies and organizational structures demonstrates the interconnected nature of the research themes. This integration is particularly relevant in high-performance sport where multidisciplinary teams with diverse expertise must rapidly coordinate decisions across medical, psychological, coaching, conditioning and tactical domains (3). While our findings align with team decision-making research in other fields (22), the sport-specific challenge of balancing support staff perspectives with the ultimate authority of the coaching staff creates unique decision-making dynamics that are not typically present in more horizontally-structured organizations.

### Methodological considerations and alternative interpretations

Our qualitative approach provided rich insights into PSLs' perceptions of decision-making. Yet there are clear research limitations. The descriptive nature of our methodology captured participants' beliefs about effective decision-making, rather than measuring actual decision-making effectiveness. Alternative interpretations of our findings might suggest that PSLs' emphasis on emotional intelligence reflects *post-hoc* rationalization, rather than actual decision-making processes; or that organizational structures may sometimes constrain, rather than enhance, decision quality in rapidly changing environments (11, 12).

Also worth considering is that, while PSLs consistently valued team decision-making, there may be circumstances where more centralized or rapid decision processes better serve performance outcomes. The balance between deliberative, inclusive decision-making and decisive leadership likely depends on contextual factors including time pressure, stakes, and the content-specific nature of the decision (3, 16).

### Synthesis and integration of findings

Collectively, our findings reveal a complex interplay between individual competencies (emotional intelligence, experience) and structural factors (organizational frameworks, team processes) in high-performance sport decision-making. The integration of these elements creates a comprehensive framework that encompasses:

i. the foundational importance of decision-making and approaches to enhancement
ii. the processes, tracking methods, and obstacle navigation strategies employed by PSLs
iii. the defining characteristics of effective decision-makers in these unique contexts

This framework not only aligns with the stated research objectives, but also offers practical guidance for developing more effective decision-making capabilities in high-performance sport environments.

### Practical implications and implementation

Our findings provide for the potential of actionable insights directly aligned with the three research objectives. The following evidence-based recommendations are organized by priority and potential impact in high-performance sporting scenarios.

#### Enhancing individual decision-making capabilities

PSLs may be served well by prioritizing the development of emotional intelligence competencies through:

- Structured self-awareness training: Implementing regular reflective practice protocols to evaluate emotional responses before making critical decisions
- Formal social awareness development: Engaging in perspective-taking exercises that enhance understanding of how decisions impact team members and stakeholders
- Relationship management skills practice: Utilising role-playing scenarios to practice difficult conversations and collaborative decision processes

Such development could enhance PSLs ability to consider their own psychological and physiological states prior to making important decisions, while also enabling them to make decisions that reflect an empathic approach to both individuals within their organisation, and the overall organisational context.

Additionally, individuals recruited into decision-making leadership roles in high-performance sport should have had adequate experience and sufficient opportunity to develop the context-specific knowledge and skills required of the position.

Furthermore, based on our findings regarding experience as a critical factor (Table 2E), we recommend:

- Experiential mentorship programs: Pairing less experienced staff with veteran decision-makers to accelerate contextual knowledge acquisition
- Scenario-based training: Implementing structured decision scenarios that simulate the complexity and pressure of real performance environments
- Formal decision-making education: Incorporating evidence-based training modules on cognitive bias, probabilistic reasoning, and decision frameworks

#### Enhancing team decision-making processes

Our findings strongly support the implementation of structured team approaches to decision-making, particularly for complex scenarios. Based on participant insights, organizations should establish:

- Formal collaborative frameworks: Creating documented processes for when and how to engage multi-disciplinary input
- Decision-making roles matrix: Clearly defining decision authority, consultation requirements, and information-sharing protocols
- Regular decision review meetings: Implementing feedback loops and review processes

These initiatives involve developing and encouraging a collaborative team environment and, through organisational structuring, establishing unambiguous roles and responsibilities, open lines of communication, and clear decision-making processes. Furthermore, facilitating group reflective practice may help performance support team members learn from past decisions and develop their decision-making competencies.

#### Implementation roadmap

Based on these findings, we recommend a sequential approach to enhancing decision-making in high-performance sport environments:

1. Begin with organizational structure development to establish clear decision-making frameworks
2. Implement emotional intelligence development for key decision-makers
3. Create experiential learning opportunities while simultaneously introducing formal training
4. Establish regular review and reflective practice protocols to continuously refine processes

Organizations implementing these research-based recommendations may experience more consistent, transparent, and effective decision-making processes leveraging both individual expertise and collective intelligence; thereby ultimately enhancing performance outcomes in high-performance sport environments.

### Limitations of this research

This research has several limitations. The sample characteristics included a small sample size (15 participants) with limited demographic diversity (12 male, 3 female) from a restricted range of professional contexts. Methodological constraints were present, as reliance on semi-structured interviews introduces potential interviewer bias. The perceptual focus of the study meant that it captured participants' perceptions of good decision-making rather than measuring actual decision-making effectiveness. A single stakeholder perspective was taken, as we only examined PSLs' viewpoints without including other team members. Finally, the context specificity of the findings may limit their generalisability to all high-performance sport environments or leadership contexts.

### Future research directions

Future research should focus on several key areas. Effectiveness assessment is needed to formally evaluate decision-making processes and outcomes using objective performance metrics. Training intervention studies should be developed and tested to provide formal decision-making training programs based on identified competencies. Multi-stakeholder investigations could explore the perspectives of support staff, coaches, and athletes to develop comprehensive decision-making frameworks. Comparative analyses would help examine approaches across different sports, organizational structures, and competitive levels. Finally, longitudinal studies are required to track how decision-making processes evolve over time and across different competitive cycles.

## Conclusion

These findings may help establish a framework for developing evidence-based interventions serving to enhance decision-making effectiveness. The identified elements of emotional intelligence, experiential knowledge, and organizational structures represent critical leverage points that may enhance leadership practices and driving measurable improvements in performance outcomes.

The findings suggest sport organizations can systematically enhance their decision-making capabilities by:

1. Implementing emotional intelligence development programs targeting self-awareness and relationship management skills
2. Creating structured mentorship and scenario-based learning opportunities that accelerate contextual knowledge acquisition; and
3. Formalizing collaborative decision protocols with clear role delineation and regular review processes.

Developing these three interdependent domains, directly supported by the themes identified in our results, simultaneously rather than in isolation, could drive transformational change in the quality and effectiveness of performance support teams decision-making. Changes which should, ultimately, translate into measurable performance gains and competitive advantages. The evidence presented here provides not just theoretical understanding, but also actionable pathways for sport organizations to elevate their decision-making; moving from intuitive to systematic; from reactive to strategic, and from individual to optimally collaborative.

Decision-making represents a cornerstone of effective performance leadership in high-performance sport. Nevertheless, research examining how these decisions are made in practice has been limited. This study sought to explore how PSLs in high-performance sport approach decision-making and understand the factors that influence their decisions.

## Data Availability

Requests to access the datasets should be directed to 15086453@studentmail.ul.ie.

## Appendix

**Table A1:** Semi-Structured interview guide.

Purpose:	Question:	Probe:	Prompts:
What do you want to know or find out?	What ‘open’ question do you need to ask to achieve this purpose?	What ‘open’ question can I ask to get info on the things I want to know if they don’t seem to understand the main question? Or if they don’t provide enough detail in their answer?	If they still don’t give me the information that I’m most interested in then what can I ask them to directly comment on?
Opener & decision-making importance	Tell me about your role?	How important is DM to your role?	How much of your success comes down to your ability to make good decisions/judgements/forecasts?
Decision-making obstacles	What are the biggest obstacles you are currently seeing to decision-making in elite sport?	Where do you feel we fail as practitioners when it comes to decision-making?	Organisational? Individual? You didn’t mention environmental factors, Bias, VUCA, Overconfidence, Experience, teams, egos, hierarchy?
Navigating difficult decisions	When faced with a difficult decision, what do you do/how do you arrive at a decision?	What enables you to be effective at making the right decision? What are the steps you take to make good decisions?	You haven’t mentioned any specific process/strategies? Do you have a set process/collecting of strategies? Within the process, do you have any specific strategies?
Traits & characteristics of good-decision-makers	What are the characteristics of good-decision-making in your environment/context?	What do they do differently than others? Who would you hold up as a good decision-maker?	Do you identify/look for top DM performers? What do you do with this information?
Collaboration	You haven’t mentioned teams/collaboration/You have mentioned teams/collaboration Talk to me about team decision-making and what comes with it?	Is there anything specific you do to help teams collaborate for better decision-making?	What is a leader's role in team decision-making?
Tracking: Learning from decision-making	How do you learn from your decision-making (good and bad)?	Are you noting & documenting information?	How are you using this information?
Tracking: Decision-making tracking	Are you tracking decision-making performance?	How do you track?	You haven’t mentioned any specific tracking strategies? How are you using this information?
Tracking: Reviewing decisions	Do you review strategies that may be directly or indirectly linked to decision-making?	How do you do this?	What do you do with this info?
Enhancing/training for decision-making	Is decision-making trainable/is it possible to enhance decision-making through learning?	If “No”, then why do you believe this or what do you believe? If “Yes”, then how would you go about this?	If “Yes”, any specific strategies?
Decision-making education	Have you ever had formal decision-making education?	If “Yes”, what? If “No”, would you consider it & why?	
Solutions	What solutions would you offer to other sporting environments/practitioners to enhance decision-making?	What about personal strategies? (can we develop the T&C of DM?) What about professional strategies? (is DM a trainable skill?) What about organizational strategies? (systems, processes, strategies)	
Any other business	Is there any other advice, comments you would like to offer that you feel has been missed in the interview that is critical to decision-making in elite/professional sport?		
